# Thiram inhibits angiogenesis and slows the development of experimental tumours in mice

**DOI:** 10.1038/sj.bjc.6600078

**Published:** 2002-03-04

**Authors:** M Marikovsky

**Affiliations:** Department of Animal Sciences, Faculty of Agriculture, Hebrew University of Jerusalem, Rehovot 76100, Israel

**Keywords:** angiogenesis, SOD-1, thiram, oxidative stress, endothelial cells

## Abstract

Thiram-tetramethylthiuram disulphide – a chelator of heavy metals, inhibited DNA synthesis and induced apoptosis in cultured bovine capillary endothelial cells. Bovine capillary endothelial cells were 10–60-fold more sensitive to thiram than other cell types. These effects were prevented by addition of antioxidants, indicating involvement of reactive oxygen species. Exogenously added Cu^2+^ impeded specifically and almost completely the inhibitory effect of thiram for bovine capillary endothelial cells. Moreover, thiram had markedly inhibited human recombinant Cu/Zn superoxide dismutase enzymatic activity (85%) *in vitro*. Moreover, PC12-SOD cells with elevated Cu/Zn superoxide dismutase were less sensitive to thiram treatment than control cells. These data indicate that the effects of thiram are mediated by inhibition of Cu/Zn superoxide dismutase activity. Oral administration of thiram (13–30 μg mouse^−1^), inhibited angiogenesis in CD1 nude mice. Tumour development is known to largely depend on angiogenesis. We found that oral administration of thiram (30 μg) to mice caused significant inhibition of C6 glioma tumour development (60%) and marked reduction (by 3–5-fold) in metastatic growth of Lewis lung carcinoma. The data establish thiram as a potential inhibitor of angiogenesis and raise the possibility for its use as therapy in pathologies in which neovascularisation is involved, including neoplasia.

*British Journal of Cancer* (2002) **86**, 779–787. DOI: 10.1038/sj/bjc/6600078 www.bjcancer.com

© 2002 Cancer Research UK

## 

Angiogenesis, the growth of new capillary blood vessels by sprouting from established vessels, is a tightly controlled process (Folkman and Klagsburn, 1987; Folkman, 1990, 1995). The hypoxic induction of angiogenesis is a hallmark of pathological processes such as wound healing and solid tumour formation. This hypoxic induction of new blood vessel formation is strongly correlated also with the disrupted circulation, the rapid proliferation and growth characteristic of these states. Angiogenesis involves intense endothelial cell cytokine-dependent proliferation and hypoxia/re-oxygenation. Cultured endothelial cells produce reactive oxygen species (ROS) spontaneously (Sundqvist, 1991; Terada *et al*, 1991) and this is augmented by hypoxia/reoxygenation (Ratych *et al*, 1987; Inauen *et al*, 1990; Lum *et al*, 1992; Zweier *et al*, 1988, 1994a,b; Terada, 1996) as well as by cytokines (Matsubara and Ziff, 1986). *In vivo*, oxygen radicals are also produced as by-products of normal oxidative metabolism (Malmstrom, 1982). Hence, proliferating cells with a higher metabolism produce more oxygen radicals. ROS have been implicated in the mechanism of damage following reperfusion of ischaemia (McCord, 1985), and vascular endothelial cells are the most vulnerable targets for free radicals produced at the time of organ reperfusion after cold preservation (Connor *et al*, 1992). Angiogenesis is a continuous process of re-oxygenation. The fact that ROS are produced by endothelial cells, especially under conditions of re-oxygenation and the very high sensitivity of endothelial cells to ROS poses a physiological need to scavenge these toxic oxygen radicals, which otherwise will lead to damage and apoptosis of the vasculature.

Cu/Zn superoxide dismutase (SOD-1) is a key enzyme in the dismutation of the potentially toxic superoxide radicals into hydrogen peroxide and dioxygen (Fridovich, 1978). We have recently shown that transgenic mice over-expressing human SOD-1 have a higher angiogenic potential than control non-transgenic mice (Marikovsky *et al*, 2002). Since angiogenesis is characterised by proliferating endothelial cells and re-oxygenation, we speculated that inhibition of SOD-1 will diminish the ability of endothelial cells to confront the increased level of ROS during angiogenesis, thus, resulting in inhibition of angiogenesis, of tumour development and metastasis. This assumption is consistent with recent findings demonstrating that the anti-angiogenic compound 2-methoxyoestradiol (Fotsis *et al*, 1994) is a SOD-1 inhibitor (Huang *et al*, 2000).

Thiram-tetramethylthiuram disulphide (TR) is a fungicide and a heavy metal chelator. Here we demonstrate that TR inhibits SOD-1 activity *in vitro*, induces apoptosis in endothelial cells *in vitro* and inhibits angiogenesis *in vivo*. Tumour growth is dependent upon the formation of new blood vessels in the tumour and its surroundings (Folkman, 1990, 1995). To determine if systemic administration of TR could inhibit an angiogenesis-dependent tumorigenesis, two models were used. TR inhibited by 60% C6 glioma tumour development and reduced by 3–5-fold the metastatic load of Lewis lung carcinoma in mouse lungs. These data suggest that TR, an inhibitor of SOD-1, may affect angiogenesis and raise the possibility for the use of TR as therapy in pathologies in which neovascularisation is involved, including neoplasia.

## MATERIALS AND METHODS

### Materials

Thiram (Sigma, St Louis, MO, USA), N-Acetyl-L-cysteine (NAC), 4,5-dihydroxy-1,3 benzene-disulphonic acid (Tiron), Glutathione (reduced) were all purchased from Sigma St Louis, MO, USA). Recombinant basic fibroblast growth factor (b-FGF) was kindly provided by Prof G Neufeld. Mouse epidermal growth factor (EGF) was purchased from Collaborative Biomedical Products (Bedford, MA, USA). Recombinant heparin-binding EGF (HB-EGF) was kindly provided by Dr Judith A Abraham (Scios Nova Inc., Mountain View, CA, USA).

### Cell lines

C6 rat glioma cells were routinely cultured in DMEM (Dulbecco's Modified Eagle's Medium) supplemented with 5% foetal calf serum (FCS, Biological Industries, Israel), penicillin (100 U ml^−1^), streptomycin (100 mg ml^−1^) (Biological Industries, Israel) and 2 mM glutamine (Biolab Ltd., Israel) (GPS) and 125 μg ml^−1^ fungizone (Biolab Ltd., Israel). The metastatic clone D122 was kindly provided by Prof L Eisenbach (Weizmann Institute, Rehovot, Israel) and was used by us for tissue culture and for *in vivo* experiments. D122 cell cultures were maintained in DMEM supplemented with 10% heat-inactivated foetal calf serum (FCS, Biological Industries, Israel), penicillin (100 U ml^−1^), streptomycin (100 mg ml^−1^) (Biological Industries, Israel), 2 mM glutamine (Biolab Ltd. Israel), sodium pyruvate (1 mM) and nonessential amino acids (Biological Industries, Israel). Brain bovine capillary endothelial cells (BCE), bovine aortic endothelial cells (BAE) and bovine vascular smooth muscle cells (BSMC) were kindly provided by Prof I Vlodavsky. BCE and BAE were cultured at 37°C in low glucose DMEM (1 g l^−1^) supplemented with 10% calf serum (HyClone, Logan, UT, USA), basic fibroblast growth factor (bFGF) (1 ng ml^−1^) and GPS. BSMC and 3T3 fibroblasts were cultured in normal glucose DMEM (4.5 g l^−1^) supplemented with 10% foetal calf serum (HyClone, Logan, UT, USA) and GPS. The BALB/MK epidermal keratinocyte cell line (kindly provided by Dr S Aaronson, National Cancer Institute, Bethesda, MD, USA), were cultured as described (Rubin *et al*, 1989; Marikovsky *et al*, 1993, 1995, 1996). Cu/Zn superoxide dismutase (SOD-1)-transfected PC12 cells were kindly donated by Prof Y Groner (Weizmann Institute, Rehovot). PC12 cells over-expressing SOD-1 (P) were transfected with a plasmid containing full length of human recombinant SOD-1. Parental PC12 cells (N) were transfected with a plasmid containing only the NEO resistant gene (control) as previously described (Elroy Stein and Groner, 1988). PC12 cells were grown in DMEM containing 10% FCS (Biological Industries, Israel) and 10% horse serum (Gibco, Grand Island, NY, USA) in presence of G418 Geneticin Sulphate (NEO) 400 μg ml^−1^ (Gibco BRL).

### Measurement of DNA synthesis

C6 rat glioma cells were plated in 96-well plates (Nunc, Denmark) (5000 cells per well) in DMEM with 5% FCS. After 6 h the cells were rinsed and incubated for 48 h in serum free medium. FCS (5%) was then added to the cells for 24 h. TR was added for 24 h at various concentrations. [^3^H]-methyl-thymidine (5 μCi ml^−1^) (ROTEM Ind. Ltd. Israel) was added to the cells for the last 14 h. BCE, BAE and BSMC were plated in 24-well plates (6000 cells per well) in 500 μl growth medium. After 24 h TR was added for 24 h at various concentrations. [^3^H]-methyl-thymidine was added for the last 6 h. DNA synthesis assay in BALB/MK keratinocytes was performed as previously described (Rubin *et al*, 1989; Marikovsky *et al*, 1993, 1995, 1996). PC12 cells were plated in 96-well plates (5000 cells per well) in 200 μl growth medium. After 24 h TR was added for 24 h at various concentrations. [^3^H]-methyl-thymidine was added for the last 6 h. All DNA synthesis assays were performed in triplicates.

TR was prepared in 10 mM stock solutions in DMSO. Control samples were incubated with the appropriate concentration of DMSO. Inhibition was calculated as percentage of DNA synthesis of control. Antioxidants were co-incubated with TR and recovery of DNA synthesis was calculated as percentage of DNA synthesis of control.

### TUNEL assay for apoptosis

Cells were cultured on microscope slides for 48 h in presence of growth media as described (Abramovitch *et al*, 1998b, 1999; Marikovsky *et al*, 1993, 1995, 1996), until reaching 30–40% confluence. TR was then added to the cells for 6 h or for 20 h in the case of BALB/MK keratinocytes. Cells were fixed with 4% paraformaldehyde. Apoptosis was analysed by the *in situ* TUNEL staining that was carried out as before (Wride *et al*, 1994). The slides were then stained with haematoxylin. Experiments were repeated twice with triplicates.

### Inhibition of SOD activity

SOD-1 activity was determined as described (Elroy Stein *et al*, 1986). Various concentrations of TR were incubated in the presence of human recombinant SOD-1 (20 ng ml^−1^). Experiments were repeated twice.

### Subcutaneous angiogenesis in nude mice

Agarose beads containing b-FGF or HB-EGF (10 μg bead^−1^). Beads were implanted subcutaneously 1 cm away from the incision site as described (Abramovitch *et al*, 1998a,b; 1995). Experiments were carried out for 4 days in CD1 nude male mice. Each day an aqueous solution with or without TR (13–30 μg mouse^−1^) was introduced per os (p.o.) to the mice using a feeding needle. Treatment was for 3 days starting from the day of bead implantation until 1 day before termination. Experimental group included four animals and experiments were repeated three times. Quantitative analysis was done by use of NIH Image 1.61 software.

### Growth of C6 glioma tumours

C6 rat glioma cells (10^6^) were injected subcutaneously into the back of the neck of CD1 nude male mice. Three days following tumour cells injection, aqueous solutions with or without TR (25–120 μg mouse^−1^) were introduced p.o. to the mice using a feeding needle. Mice were treated three times per week. Mice were sacrificed 30 days following C6 cells injection by injecting 20 mg mouse^−1^ Xylazine (i.p.) and tumours were removed, weighed, fixed in buffered formalin and histological sections were prepared and stained with Haematoxylin-Eosin and light green. Blood vessels were stained with the endothelial-specific Bandeirea simplicifolia agglutinin-I (GSL) (Sigma Inc., St Louis, MO, USA) as described (Alroy *et al*, 1987). Blood vessels count was the average of five different ×400 fields. Each experimental group included eight animals, and experiments were repeated twice.

### Metastasis of Lewis lung carcinoma tumours to the lungs

The Lewis lung carcinoma (3LL), which originated spontaneously in a C57/BL/6J(H-2^b^) mouse, is a malignant tumour that produces spontaneous lung metastases. The metastatic clone D122, was kindly provided by Prof L Eisenbach, and was used by us for tissue culture and for *in vivo* experiments.

D122 cells (5×10^5^) were injected i.v. to the tail of C57/BL male mice (8–10 weeks old) and TR (13–40 μg mouse^−1^) or water were administered p.o. three times per week and was started 3 days following tumour cell injection. Mice were sacrificed 24 days following injection of D122 cells by injecting 20 mg mouse^−1^ Xylazine (i.p.) and lungs were weighed. Each experimental group included eight animals, and experiments were repeated twice.

The animal ethics in all animal experiments meet the standards required by the UKCCCR Guidelines for the welfare of animals in experimental neoplasia (second edition), as stated in the ‘Instructions to Authors’ forms.

## RESULTS

### Inhibition of capillary endothelial cell proliferation *in vitro* by TR

**Figure 1:**
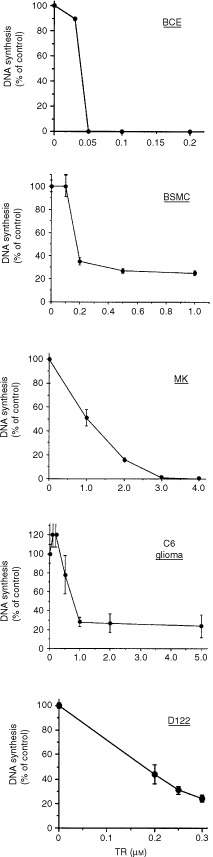
TR inhibits DNA synthesis in various cell types. DNA synthesis was measured by the incorporation of [^3^H]-thymidine into the cells, as described in Materials and Methods. TR at various concentrations was incubated with BCE, BSMC, BALB/MK, C6 rat glioma cells and with D122 cells as described in Materials and Methods. TR inhibited in a dose-dependent manner DNA synthesis in various cell types. Experiments were done in triplicates and inhibition was calculated as percentage of DNA synthesis of non-treated control.

We have examined the effect of TR on the growth of capillary endothelial cells *in vitro*. DNA synthesis in BCE cells was measured in the presence of increasing concentrations of TR incubated for 24 h with the cells. TR was able to inhibit DNA synthesis in BCE cells in a dose dependent manner (Figure 1

). Complete inhibition of DNA synthesis was shown at 50 nM TR (Figure 1). BAE cells demonstrated similar results to those of BCE (not shown). Both endothelial cell types were 10–60-fold more sensitive to the inhibitory effect of TR on DNA synthesis than other cell types such as BALB/MK keratinocytes, C6 rat glioma cells, D122 Lewis lung carcinoma or BSMC. The inhibitory effect of TR was irreversible (not shown).

### Inhibition of capillary endothelial cell proliferation* in vitro* by TR is reversed by copper

TR is a chelator of heavy metals. We have examined the possibility that its inhibitory effect for endothelial cells is via this characteristics. Exogenously added Cu^2+^ abolished the inhibitory effect of TR for BCE by preincubating the reagent with 2 μM Cu^2+^ (Table 1

**Table 1:**
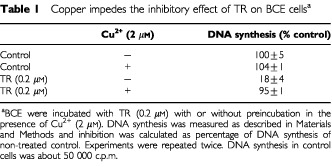
Copper impedes the inhibitory effect of TR on BCE cells^a^

), but not with other metals such as Zn^2+^, Ni^2+^, Mn^2+^ or Fe^2+^ (not shown). Copper alone, at 2 μM, had no effect on BCE.

### TR inhibits superoxide dismutase activity *in vitro*

**Figure 2:**
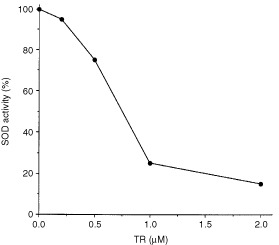
TR inhibits SOD-1 activity *in vitro*. Various concentrations of TR were incubated with 20 ng ml^−1^ of human recombinant SOD-1. SOD-1 activity was measured as described in Materials and Methods and calculated as per cent from the activity of control non-treated 20 ng ml^−1^ SOD-1. TR had markedly inhibited human recombinant SOD-1 enzymatic activity (85%). Experiments were repeated twice.

Cu/Zn superoxide dismutase (SOD-1) is a protective enzyme responsible for maintaining lower levels of superoxide radicals within the cell (Fridovich, 1978). Copper is essential for the enzymatic activity of SOD-1. Following the observation that copper reversed the inhibitory effect of TR on endothelial cells, we thought that TR effect might be via SOD-1. At concentrations similar to those used in the biological assays, TR had markedly (85%) and in a dose dependent manner inhibited pure recombinant human SOD-1 enzymatic activity (Figure 2

). It is possible thus, that TR inhibits endothelial cell growth by inhibiting SOD-1 through the chelation of its copper moiety.

### Antioxidants prevent the inhibitory effect of TR for endothelial cells

**Figure 3:**
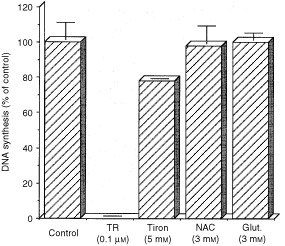
Antioxidants prevent the inhibitory effect of TR on endothelial cells. N-acetylcysteine: NAC (3 mM), glutathione: Glut. (3 mM) and Tiron (5 mM) were co-incubated with 0.1 μM of TR for 24 h and DNA synthesis was measured as described in Materials and Methods. The effect of antioxidants was calculated as per cent of DNA synthesis compared with that in control cells treated with antioxidants alone. Inhibition of DNA synthesis in BCE by TR was fully restored by co-incubation with antioxidants. DNA synthesis in control cells was about 50 000 c.p.m.

Inhibition of SOD-1 activity may alter the delicate balance of ROS within the cell. We therefore examined the effect of antioxidants on TR inhibition. Addition of the antioxidants: N-acetyl cysteine (NAC), reduced glutathione and Tiron (a selective scavenger of O_2_^-^) significantly reversed the inhibitory effect of TR (Figure 3

). This indicates that the inhibitory effect of TR on endothelial cells involves increase in production of ROS. Interestingly, exogenously added human recombinant SOD-1 did not affect the inhibition induced by TR indicating that TR effect is intra-cellular and that the ROS function internally.

### PC12** cells over-expressing SOD-1 are more resistant to inhibition by TR

**Figure 4:**
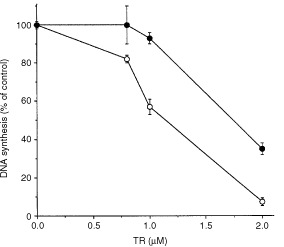
Over-expression of SOD-1 in PC12 cells renders them more resistant to the inhibitory effect of TR. Parental non-transfected (open circle) and SOD-1-transfected (solid circle) PC12 cells were incubated in the presence of various concentrations of TR, and DNA synthesis was measured as described in Materials and Methods. The effect of TR was calculated as per cent of DNA synthesis compared with that in control cells not treated with TR. PC12 cells transfected with human SOD-1 were more resistant to the inhibitory effect of TR than parental non-transfected PC12 cells. DNA synthesis in PC12 non-treated cells was about 30 000 c.p.m.

SOD-1 – an important ROS scavenger – is essential for the survival of proliferating cells. The data presented in Figures 2 and 3 demonstrate that TR: (1) inhibits the enzymatic activity of SOD-1 and (2) increases ROS level in BCE cells as a result of SOD-1 inhibition to dismutate superoxide into H_2_O_2_, indicate that TR inhibits SOD-1 in BCE cells. To further support the notion that over-expression of SOD-1 is a general mechanism to confer resistance to TR we reasoned that any cell type expressing higher levels of SOD-1 will better tolerate the inhibitory effect of TR. Stably transfected PC12 cells that over-express SOD-1 (two-fold) (Elroy Stein and Groner, 1988), were treated with TR and DNA synthesis was monitored in comparison to parental cells. As shown in Figure 4

proliferating PC12 cells, which elevated SOD-1, were less sensitive to the inhibitory effect of TR than the corresponding parental cells. This result further supports the notion that the effects of TR are mediated via inhibition of SOD-1.

### TR induces apoptosis in cultured endothelial cells

**Figure 5:**
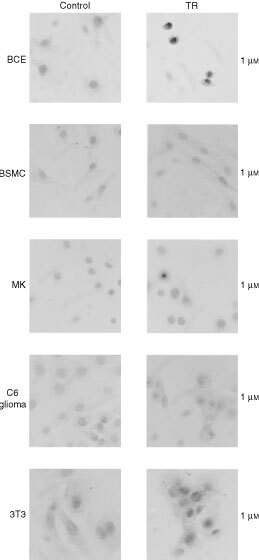
TR induces apoptosis in endothelial cells. BCE, BSMC, BALB/MK keratinocytes, C6 glioma cells and 3T3 fibroblasts following incubation with the indicated concentrations of TR for 6 h or for 20 h with BALB/MK, were analysed by the TUNEL method as described in Materials and Methods. Only the nuclei of treated BCE were labelled using the TUNEL staining method. The nuclei of treated BSMC, BALB/MK C6 glioma cells and 3T3 fibroblasts were not labelled. Experiments were repeated twice with triplicates.

To further characterize the mechanism by which TR inhibits BCE cell growth, treated BCE cells were assayed for induction of apoptosis by the TUNEL method. BCE cells (40–50% confluence) that were incubated for 6 h with 1 μM of TR underwent apoptosis as evident by the positive TUNEL signals (Figure 5

). In contrast, TR did not induce apoptosis in several other cell types: BSMC (1 μM), 3T3 fibroblasts (1 μM), C6 rat glioma cells (5 μM) and BALB/MK keratinocytes (5 μM) (Figure 5). Capillary endothelial cells are, thus, distinct in their apparent sensitivity to the TR-induced apoptosis. Typically for cells undergoing apoptosis, endothelial cells treated with TR, quickly became rounded and later on detached from the plate. In contrast, the other cell types examined did not change their shape when treated with TR. Interestingly, confluent, quiescent endothelial cells treated with TR (0.3–3 μM) did not change their shape to the rounded form or became detached, neither was their DNA synthesis affected (not shown). This may suggest that TR affects endothelial cells in a cell cycle-dependent manner. Supporting results were obtained by FACS analysis (not shown). DNA content analysis of BCE cells incubated with 0.3–5 μM of TR demonstrated appearance of a significant sub-diploid apoptotic population of cells. Most of the cells that were not in the apoptotic population, were found in the G_0_/G_1_ phase (not shown).

### Inhibition of *in vivo* neovascularisation by TR

**Figure 6:**
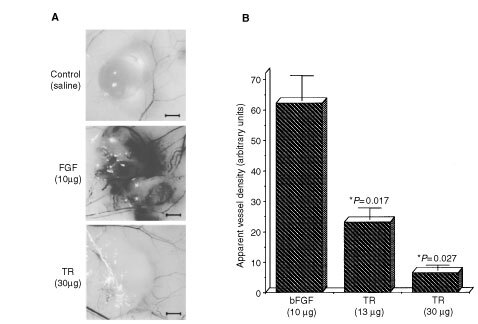
TR inhibits neovascularisation in CD1 mice. Agarose beads containing bFGF (10 μg bead^−1^) were implanted subcutaneously as described in Materials and Methods. (**A**) The angiogenic potential of bFGF *in vivo* is demonstrated 4 days after implantation in skin specimens. TR (13–30 μg mouse^−1^ day^−1^) was introduced p.o. every day starting with bead implantation. TR inhibited almost completely in a dose dependent manner neovascularisation inside and around the bead. Bar is 1 mm. (**B**) Quantitative analysis of blood vessel density around and inside the beads. Quantitative analysis was done by use of NIH Image 1.61 software. TR at 13–30 μg mouse^−1^ day^−1^ inhibited angiogenesis by 80–95% respectively (*n*=4).

Following the observation that TR induces apoptosis in endothelial cells, we examined the effect of TR on angiogenesis. Basic FGF was implanted subcutaneously into CD1 nude mice. After 4 days new blood vessels clearly developed around and inside the beads containing the angiogenic factor (Figure 6A

). Blood vessel formation around the FGF beads was very intense and seems very dense since it reflects the various layers of vessels in the bead. The saline-containing control beads appeared on the other hand, clear and without any new blood vessels being formed around or within the beads (Figure 6A). Significantly, when mice were fed daily p.o. during 3 days with 30 μg mouse^−1^ day^−1^ of TR, angiogenesis around and inside the bFGF-containing beads was markedly reduced (Figure 6A). Quantitative analysis of the apparent blood vessel density around and inside the beads demonstrates a dose dependent inhibition of angiogenesis (Figure 6B). TR at 13–30 μg inhibited angiogenesis by 80–95% respectively. Similar results were obtained when HB-EGF which induces expression of vascular endothelial growth factor (VEGF) (Abramovitch *et al*, 1998b) was used instead of bFGF (not shown). The data show that TR inhibits induced angiogenesis and that the inhibition is not restricted to one specific inducer. Visual examination of various tissues (kidney, liver, stomach, lungs and spleen) of TR treated CD1 mice as well as histological sections prepared from these tissues revealed no pathological findings in the tissues or in their already established blood vessels.

### TR inhibits C6 glioma tumour growth *in vivo*

TR inhibited neovascularisation *in vivo* and C6 glioma cell growth in culture. Since active angiogenesis is essential for the progressive growth of solid tumours beyond a diameter of a few millimetres (Folkman, 1990), we examined whether systemic treatment with TR will slow tumour development. TR at concentrations that caused inhibition of angiogenesis *in vivo*, significantly retarded C6 glioma tumour growth in CD1 nude mice. The lower concentration (25 μg mouse^−1^) was more effective than the higher dose (120 μg mouse^−1^) (Table 2

**Table 2:**
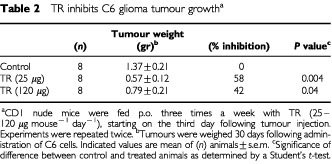
TR inhibits C6 glioma tumour growth^a^

). This finding is consistent with the observation that high concentrations of TR were less inhibitory for cell growth (not shown). This may be due to polymerisation of TR at higher concentration. Since TR inhibits both angiogenesis *in vivo* and C6 in culture, it is possible that tumour growth inhibition may have resulted from inhibition of neovascularisation combined with inhibition of tumour cell growth. Histological sections prepared from C6 tumours and stained for Hematoxylin-Eosin and light green demonstrated a clear difference in the appearance of C6 cells in tumours from TR-treated animals *vs* C6 cells in tumours from control non-treated animals (Figure 7

**Figure 7:**
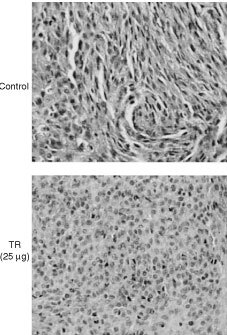
Histological sections of C6 tumours stained with Haematoxylin-Eosin and light green. C6 tumours from TR-treated animals are different from C6 tumours from control non-treated animals. Cells in tumours from control non-treated animals have a more pleomorphic appearance, with a bizarre form. Cell size and shape in tumours from TR-treated (TR 25 μg) or (TR 120 μg) animals are more uniform and are more differentiated (nuclei stain and shape). Magnification (×120).

). Cells in tumours from control non-treated animals have a more pleomorphic appearance, with a bizarre form. The tumour is made of a mixture of small and large cells, most of them undifferentiated with various orientations and polarisation. Cell size and shape in tumours from TR-treated animals are more uniform and are more differentiated (nuclei stain and shape).

**Figure 8:**
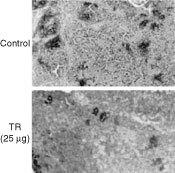
TR reduces blood vessel number in C6 tumours. Histological sections of C6 glioma tumours were stained for Haematoxylin-Eosin and light green followed by staining with the endothelial-specific GSL lectin (dark blue staining) as described in Materials and Methods. The number of blood vessels per field in C6 tumours derived from mice treated with TR (25 μg) was reduced by 33% compared with non-treated control (10.8±0.41 s.e. and 16.1±0.47 s.e., accordingly (*P*<0.0003).

Visual examination of various tissues (kidney, liver, stomach, lungs and spleen) of TR treated CD1 mice as well as histological sections prepared from these tissues revealed no pathological findings in the tissues or in their already established blood vessels. However, histological sections of C6 glioma tumours stained with the endothelial-specific GSL lectin have demonstrated that the number of blood vessels per field (×400) in C6 tumours derived from mice treated with TR (25 μg) was reduced by 33% compared with non-treated control (10.8±0.41 s.e. and 16.1±0.47 s.e., accordingly (*P*<0.0003)) (Figure 8

).

### TR inhibits Lewis lung metastasis

We next examined the effect of TR on metastatic growth using the Lewis lung carcinoma model in C57/BL mice, using concentrations that were shown to be inhibitory for angiogenesis *in vivo*.

**Figure 9:**
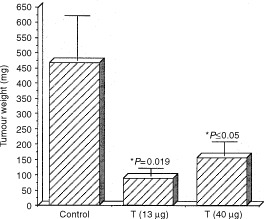
TR inhibits Lewis lung metastatic growth in the lungs. D122 tumour cells were injected into C57/BL mice and 3 days later TR (13–40 μg mouse^−1^) was introduced p.o. three times a week. Twenty-four days following tumour injection, the lungs were weighed. Metastatic growth in the lungs of TR-treated animals, compared with that in water-fed control was significantly smaller (*P*=0.019–0.05) (*n*=6). At 13–40 μg mouse^−1^ TR decreased metastatic growth in the lungs 5–3-fold accordingly. Experiments were repeated twice. The weight of lungs derived from normal mice was subtracted from that of the metastasised lungs. *P* by student's *t*-test.

We have used the i.v. model. Three days following D122 tumour cell injection, TR was introduced p.o. three times per week, at concentrations of 13–40 μg mouse^−1^ and the growth of metastatic foci in the lungs was inhibited by 83–68% accordingly (Figure 9

). Since TR inhibits both angiogenesis *in vivo* and D122 cells in culture, it is possible that inhibition of metastatic foci growth may have resulted from inhibition of neovascularisation combined with inhibition of tumour cell growth.

## DISCUSSION

Angiogenesis is associated with intense endothelial cell cytokine-dependent proliferation and hypoxia/re-oxygenation. Endothelial cells produce ROS in response to cytokines (Matsubara and Ziff, 1986) and to hypoxia/re-oxygenation (Ratych *et al*, 1987; Inauen *et al*, 1990; Lum *et al*, 1992; Zweier *et al*, 1988, 1994a,b; Terada, 1996). ROS have been implicated in the mechanism of damage following ischaemia and during low-flow hypoxia (McCord, 1985). Accumulated evidence has shown that vascular endothelial cells are the most vulnerable targets for free radicals produced at the time of organ reperfusion after cold preservation (Connor *et al*, 1992) and that preservation of endothelial function is associated with a reduction in superoxide radical generation (Mann *et al*, 1997).

SOD-1 is a protective enzyme responsible for maintaining lower levels of superoxide radicals within the cell (Fridovich, 1978; Bannister *et al*, 1987; Crapo *et al*, 1992). Previous studies have shown that down-regulation of SOD-1 activity induces apoptosis of neuronal cells (Troy and Shelanski, 1994) and that up-regulation of SOD-1 by shear stress is an important mechanism preserving the integrity of the endothelium after pro-apoptotic stimulation (Dimmeler *et al*, 1999). In addition, we have recently shown that transgenic mice over-expressing human SOD-1 demonstrate an increased angiogenic potential compared to control parental non-transgenic mice (Marikovsky *et al*, 2002). Alterations in the activity of SOD-1 may therefore affect angiogenesis and angiogenesis-dependent pathologies.

The data presented in this study identify TR as an effective inhibitor of angiogenesis. Oral administration of TR, which was shown here to inhibit SOD-1, almost completely abrogated the formation of new blood vessels. The ability of TR to inhibit at low concentrations the growth of cultured capillary endothelial cells and to specifically induce apoptosis in endothelial cells unlike other cell types examined, suggests that the drug acts directly on capillary endothelial cells. Interestingly, this sensitivity was found predominantly in proliferating endothelial cells. Systemically administered TR specifically abrogated the formation of new blood vessels, while no evidence for damage in other tissues or in existing blood vessels was observed.

Only a few specialized cell types, notably activated neutrophils and activated endothelial cells, are currently known to produce large quantities of ROS (Ryan and Vann, 1988). Increase in intracellular ROS as well as exposure to extra-cellular source of ROS (Shaikh *et al*, 1997; Suzuki *et al*, 1997) induce apoptosis in endothelial cells. One potential source of ROS is xanthine oxidase (XO) which was shown to generate free radicals in endothelial cells (Zweier *et al*, 1994a). XO was found to be much higher in capillary endothelial cells (Jarasch *et al*, 1986). Moreover, hypoxia was shown to injure endothelial cells by increasing xanthine oxidase activity (Terada *et al*, 1992)**.** Another mechanism generating ROS that could preferentially affect endothelial cells has recently been identified (Beckman *et al*, 1990). It shows that superoxide and nitric oxide can react rapidly to form peroxynitrite, a potent ROS. Since endothelial cells produce both superoxide and nitric oxide, they may be particularly vulnerable to oxidant injury by this mechanism.

TR is a potent chelator of heavy metals. Chelation of enzymes' metal moiety may lead to loss of activity. Copper is essential for the enzymatic activity of SOD-1. We have shown that TR strongly inhibits SOD-1 activity and that exogenously added copper and not Zn^2+^, Ni^2+^, Fe^2+^ or Mn^2+^, was able to out-compete the inhibitory effect of TR on endothelial cell growth. Inhibition of SOD-1 by TR could decrease the capacity of endothelial cells to scavenge the increased level of superoxides, resulting in growth arrest, apoptosis, and inhibition of angiogenesis. These data are in good agreement with recent findings demonstrating that another SOD-1 inhibitor (Huang *et al*, 2000) – 2-methoxyoestradiol is also anti-angiogenic (Fotsis *et al*, 1994). No damage was observed in already established (non-proliferating) blood vessels in the treated animals. This observation is in accordance with our observation that TR does not affect quiescent non-proliferating endothelial cells.

TR was previously shown to inhibit also other enzymes such as aldehyde dehydrogenase, aldehyde oxidase and rat lipoprotein lipase activity in adipose tissue (Freundt and Netz, 1977; Sanny and Weiner, 1987; Sadurska and Boguszewski, 1993). None of these enzymes is copper dependent. Our data is consistent with previous findings demonstrating copper ions are involved in the sequence of events leading to angiogenesis (Raju *et al*, 1982; McAuslan *et al*, 1983), that cornea colonised by capillaries induced by an angiogenic factor become rich in copper ions (Ziche *et al*, 1982; Gullino, 1986), and that depletion of copper by diet or by penicillamine inhibited brain tumour angiogenesis and growth (Brem *et al*, 1990a,b; Yoshida *et al*, 1993, 1995). It cannot be excluded that chelation of copper by TR may exert an anti-angiogenic effect in addition to SOD-1 inhibition.

We showed that antioxidants such as NAC and Tiron have significantly reversed the TR-mediated inhibition of cell growth, indicating an involvement of oxidative stress in this process. In addition, transfected PC12 cells over-expressing SOD-1, were more resistant to cell growth inhibition by TR than the parental non-transfected cells. This result further indicates that SOD-1 is involved in the inhibitory effect of TR on endothelial cell growth.

Previous results, which demonstrated reduction of spontaneous occurrence of tumours by TR, are consistent with our findings. TR was shown to reduce the incidence of spontaneous leukaemia, mammary fibroadenoma and skin masses and to slightly reduce pituitary and thyroid tumours in rats (Takahashi *et al*, 1983; Hasegawa *et al*, 1988; Maita *et al*, 1991). This reduction could be partly attributed to its anti-angiogenic activity. TR was shown to be non-clastogenic and non-carcinogenic in rats (Hasegawa *et al*, 1988) and in the skin in male Swiss albino mice (George and Kuttan, 1995).

Angiogenesis is a tightly controlled process. Nevertheless, many pathologies are driven by persistent unregulated neovascularisation. Since C6 glioma tumour development as well as lung metastasis are angiogenesis-dependent (Plate *et al*, 1992; Abramovitch *et al*, 1995; Ikeda *et al*, 1995; Niida *et al*, 1995), it is conceivable that tumour development in both models is affected by TR. Indeed, TR significantly reduced both Lewis lung metastatic growth and C6 tumor development *in vivo*. The use of an orthotopic model would be more clinically relevant and may involve different mechanisms of angiogenesis. The inhibiting effect of TR in both tumour models was achieved when administered systemically p.o. at low concentrations, similar to those that effectively inhibited angiogenesis *in vivo*. TR was inhibitory *in vitro*, for endothelial and for C6 glioma cells as well as for Lewis lung carcinoma cells. It is possible thus, that TR inhibition of tumour development and metastasis is mediated through a combined effect on both angiogenesis and tumour cell growth.
